# Uncovering intensity trajectory patterns and their association with blood pressure adaptation following school-based high-intensity interval exercise in adolescents: Wroclaw PEER-HEART study

**DOI:** 10.3389/fspor.2026.1872708

**Published:** 2026-07-03

**Authors:** Jarosław Domaradzki, Dawid Koźlenia

**Affiliations:** Faculty of Physical Education and Sport, Wroclaw University of Health and Sport Sciences, Wrocław, Poland

**Keywords:** adolescents, blood pressure, high-intensity exercise, individual response, intensity trajectory patterns, responders and non-responders, school-based intervention

## Abstract

**Introduction:**

This study examined intensity patterns during school-based high-intensity interventions and their association with blood pressure (BP) adaptation in adolescents.

**Methods:**

A total of 145 students aged 16 (69 boys) participated in either traditional HIIT or a plyometric variant (HIPT) over eight weeks (16 sessions). Exercise intensity was calculated using the heart rate reserve method. Outcomes of interest were systolic and diastolic blood pressure (SBP, DBP, respectively). Blood pressure responders (Rs) and non-responders (NRs) were identified using a Bayesian approach.

**Results:**

Latent class mixed models revealed three distinct intensity trajectories: Class 1 (low-intensity declining trajectory), Class 2 (moderate-intensity declining trajectory), and Class 3 (high-intensity U-shaped trajectory). Although no significant unadjusted association was found between responder status and trajectory class, logistic regression indicated that individuals in Class 2 had higher odds of being classified as SBP responders (OR = 3.86, *p* = 0.04), whereas Class 3, male sex, and HIPT participation were associated with lower odds of meeting the responder criterion. In contrast, linear regression analyses demonstrated that participants in Classes 2 and 3 exhibited greater mean reductions in both SBP and DBP than those in Class 1 (*p* < .01), highlighting that responder classification and the magnitude of blood pressure change reflect different aspects of adaptation. Trajectory class was the strongest predictor of BP change. HIPT was associated with greater DBP reductions (*p* = .006), whereas sex was not a significant predictor in the continuous models.

**Discussion:**

These findings indicate that adolescents may follow different patterns of exercise intensity across a school-based intervention and that these patterns may be associated with blood pressure adaptation. Monitoring session-by-session exercise intensity may therefore provide information beyond the average intensity of the entire program. However, further studies are required before trajectory-based monitoring can be translated into specific recommendations for school practice.

## 1 Introduction

Elevated blood pressure (BP) in adolescence is an increasingly common concern and a well-recognized early marker of future cardiovascular risk (1). It is one of the primary modifiable risk factors for cardiovascular disease (CVD) morbidity and mortality in the United States and a public health concern worldwide (2, 3). School-based physical activity interventions, particularly high-intensity interval training (HIIT), have shown promise in improving cardiometabolic health, including reductions in resting systolic (SBP) and diastolic blood pressure (DBP) (4, 5). These approaches are time-efficient, feasible during physical education (PE) lessons, and effective even with low training volumes (6, 7).

Recent studies have highlighted significant inter-individual variability in responses to exercise interventions, particularly high-intensity interval training (HIIT). While group-level effects of HIIT and HIPT have been reported in youth populations, a growing body of evidence suggests considerable inter-individual variability in physiological responses to exercise interventions (8, 9). This variability has been documented in diverse populations, including overweight/obese women and adolescents (10). Some individuals experience clear health benefits (responders), while others show no meaningful change (non-responders), or even adverse effects. The concept of interindividual variability in response to exercise training (IVRET) underscores that, despite statistically significant improvements at the group level—such as reductions in body fat mass—some individuals may exhibit minimal or no response to the intervention (11–13). Recognizing and understanding this variability is critical for the development of more personalized and effective training strategies. Factors such as sex, biological maturation, and initial fitness levels may influence individual responses (10). However, the classification of individuals as “responders” or “non-responders” based on arbitrary thresholds has been criticized, as it can be biased by within-subject variability and differences in mean responses (12). To address this variability and develop more personalized interventions, researchers suggest using multiple outcome measures and considering the standard deviation of individual responses (8, 12).

To date, most studies have focused on aggregate measures of training intensity, such as average heart rate or sessional training impulse (e.g., TRIMP). TRIMP is a metric used in sports science to quantify the physiological stress of a training session by combining duration and intensity, primarily based on heart rate data. It helps coaches and athletes understand the training load and make informed decisions about training planning and recovery (14, 15). However, far less attention has been paid to the trajectory or temporal structure of intensity across a multi-week training program. It is plausible that not just how hard adolescents train, but how intensity evolves over time—whether progressively, inconsistently, or following a U-shaped pattern—may influence the adaptive response of the cardiovascular system. In school-based physical education, one of the most practical approaches to evaluating exercise intensity is continuous heart rate (HR) monitoring throughout the entire lesson. This measurement reflects the internal training load or exercise dose. The long-term physiological effects of implementing high-intensity interval training (HIIT) during physical education classes can be assessed by tracking changes in systolic blood pressure (SBP), diastolic blood pressure (DBP), body weight, or cardiorespiratory fitness (CRF). These outcomes can then be used to determine the body's response relative to the exercise dose received. While previous research has explored dose–response relationships between various forms of physical activity and outcomes such as body fat, blood pressure, and CRF, such studies were typically conducted under controlled laboratory conditions rather than in real-world school environments (16–18).

Most studies evaluating school-based high-intensity exercise use summary measures such as mean heart rate or cumulative training load, which do not capture how exercise intensity changes across successive sessions. Although HIIT is characterized by alternating periods of high-intensity exercise and recovery within an individual session (19), the longitudinal pattern of intensity across a multi-week intervention may differ considerably between participants. Some individuals may maintain a relatively stable intensity, whereas others may exhibit declining, ascending, or non-linear trajectories. Such patterns may reflect differences in exercise tolerance, engagement, pacing, fatigue, or recovery across the intervention (20–22).

Participants with similar average exercise intensity may therefore experience substantially different temporal distributions of the training stimulus, potentially contributing to differences in physiological adaptation. Supervised and structured school-based interventions may support the maintenance of exercise intensity, although previous research has primarily focused on intervention effectiveness rather than session-by-session intensity trajectories (23). While blood pressure responses to exercise vary considerably among adolescents, it remains unclear whether this variability is associated with the pattern of intensity maintained throughout the intervention. To our knowledge, session-by-session intensity trajectories and their relationship with blood pressure adaptation have not been systematically examined in school-based high-intensity exercise programs.

Most school-based exercise studies describe training intensity using an average value calculated across individual sessions or the entire intervention. Although useful, an average value may conceal important differences in how exercise intensity changes over time. For example, two students may achieve a similar mean intensity despite one maintaining a relatively stable effort and the other showing a marked decline across successive sessions. Identifying these longitudinal patterns may help determine whether the way in which exercise intensity is maintained throughout a program is associated with blood pressure adaptation. This information may be relevant for the future development of feasible monitoring strategies in school-based exercise program.

Therefore, the general aim of this study was to investigate distinct patterns of training intensity progression during a school-based HIIT and HIPT program and whether the patterns are associated with individual variability in blood pressure adaptation among adolescents. Specifically, the aims were fourfold: (1) to classify participants as responders (R), non-responders (NR), or uncertain responders based on changes in resting systolic and diastolic blood pressure using a Bayesian-based threshold that accounts for individual measurement error; (2) to identify latent trajectory patterns of training intensity across 16 sessions using a data-driven latent class mixed modeling (LCMM) approach; (3) to examine the relationship between trajectory patterns and blood pressure response categories: (a) by analyzing the distribution of responders and non-responders across trajectory classes (e.g., via chi-square tests), (b) by modeling the probability of being a responder based on trajectory class, sex, and training form using generalized linear modeling (GLM); (4) To assess whether the magnitude of blood pressure change (*Δ*SBP and *Δ*DBP) is associated with trajectory class, sex, and training form using linear models.

It was hypothesized: H_1_: Adolescents participating in the intervention will exhibit heterogeneous longitudinal exercise-intensity trajectories rather than a single common pattern across all participants.; H_2_: Participants exhibiting certain trajectory patterns—particularly progressive or adaptive increases in training intensity—are more likely to demonstrate favorable blood pressure adaptations (i.e., be classified as responders); H_3_: the probability of being a responder differs significantly between trajectory classes, suggesting that not only the amount but also the structure and progression of training intensity influences cardiovascular outcomes in youth.

## 2 Materials and methods

### 2.1 Wroclaw PEER-HEART project

This study was supported by the Polish Ministry of Science and Higher Education through the “Science for Society II” program (grant no. NdS-II/SP/0521/2023/01). It extends prior research that investigated the physiological effects of two high-intensity interval training formats—HIIT and HIPT—among adolescents (24). The earlier publication outlines the full methodology, including participant recruitment, study design, and intervention procedures. For clarity, only a brief overview of these elements is provided here.

### 2.2 Ethics statement

The study protocol was approved by the Ethics Committee of the Wroclaw University of Health and Sport Sciences (approval no. 33/2018, dated 31 October 2018). All procedures involving human participants were conducted in accordance with the ethical standards set forth in the Declaration of Helsinki and the guidelines of the World Medical Association. Written informed consent was obtained from all participants and from their parents or legal guardians prior to inclusion in the study.

### 2.3 Study design and participants

An *a priori* power analysis was conducted using G*Power 3.1 (25) for the parent randomized controlled trial, based on a MANOVA design including two training modalities (HIIT, HIPT), two sexes, intervention and control groups, and three measurement occasions (baseline, post-intervention, and follow-up). Assuming a small effect size (*f* = 0.20), *α* = 0.05, and 95% statistical power, the minimum required sample size was estimated at 310 participants. A total of 307 adolescents were enrolled in the trial, with no adverse events reported during the intervention. Informed consent was obtained from all students, their parents or legal guardians, and school officials.

The present study represents a secondary analysis of the intervention arm of the trial and was restricted to participants with complete session-by-session exercise-intensity data. Accordingly, the analytical sample consisted of 145 participants: 24 boys in the HIPT group, 45 boys in the HIIT group, 46 girls in the HIPT group, and 30 girls in the HIIT group.

### 2.4 Measurements

Body height was measured to the nearest 0.1 cm using an anthropometer (GPM Anthropological Instruments, DKSH Ltd, Switzerland). Body weight and body composition were assessed using a segmental body composition analyzer (Tanita InnerScan V, model BC-601; Tanita Co., Tokyo, Japan). Body mass index (BMI) was calculated as weight in kilograms divided by height in meters squared (kg/m^2^).

Blood pressure was measured using an Omron BP710 automatic monitor (Omron Healthcare, Inc., Hoffman Estates, IL, USA) following established protocols (26). Cuff sizes were selected according to each participant's upper arm circumference to ensure accuracy. Prior to measurement, participants remained seated at rest for 10 min. Three readings were then taken at 10 min intervals, and the mean value was used for analysis. Measurements were collected at baseline, immediately post-intervention, and during the follow-up phase.

### 2.5 Intervention protocol

The intervention was implemented during regularly scheduled physical education (PE) classes, occurring twice weekly over an eight-week period. The HIIT condition followed a modified Tabata format, with progressive increases in training volume across the program. Exercise intensity was continuously monitored using Polar Verity Sense devices (Polar Electro, Finland), targeting approximately 75% of each participant's estimated maximum heart rate (HR_max), derived from the 20 m shuttle run test (27).

Each session began with a standardized 10 min warm-up, followed by structured intervals of high-intensity effort. The number of intervals increased progressively throughout the intervention: 4 intervals (20 s work/10 s rest) in weeks 1–2, 6 intervals in weeks 3–4, and 8 intervals in weeks 5–8.

The two intervention groups differed in exercise modality. The HIPT group performed primarily plyometric movements (e.g., burpees, squat jumps, mountain climbers), while the HIIT group engaged in non-plyometric, bodyweight exercises (e.g., squats, push-ups, sit-ups).

### 2.6 Intensity of the effort during session

Exercise intensity for each session (16 sessions, 2 sessions a week) was calculated using the heart rate reserve (HRR) method, according to the Karvonen formula (28):

Intensity=(HR_average−HR_rest)/(HR_max−HR_rest).

This approach expresses relative intensity as a percentage of the individual's heart rate reserve, providing a more accurate estimation than simple percentage of maximum heart rate.

### 2.7 Bayesian classification of training response

To identify individual training responses, a Bayesian analytical framework was applied, following the approach described by Maturana et al. (29). This method was selected due to the small sample size in the overweight/obese subgroup (*n* = 15 vs. *n* = 130 in the normal-weight group) and its suitability for unbalanced data and detecting potential floor effects.

For each participant, a normal distribution was modeled around the observed change score, incorporating a 5.6% baseline-derived measurement uncertainty, following the framework proposed by Mattioni Maturana et al. (29). An 89% Highest Density Interval (HDI) was calculated using the 5.5th and 94.5th percentiles of the simulated distribution. Participants were classified as follows based on the overlap of the HDI with a Region of Practical Equivalence (ROPE; ±0.05):

- Responder: HDI entirely below the ROPE (clear reduction),
- Non-responder: HDI entirely above the ROPE (clear increase),
- Uncertain: HDI overlapping the ROPE (ambiguous change).

This approach accounts for both individual variability and measurement precision, enhancing the detection of meaningful physiological responses and potential floor effects in those with low baseline values.

### 2.8 Statistics

Using a Bayesian approach, participants were classified as responders, non-responders, or uncertain. The frequencies for each group were calculated. Three classification types were compared: (1) the primary three-categories type (responder, non-responder, uncertain); (2) a more detailed four-categories type, where uncertain cases were further divided into R-like (*Δ* < 0) and NR-like (*Δ* ≥ 0); and (3) a binary two-categories type, in which R-like individuals were grouped with responders and NR-like with non-responders. A Next, all three classification types (two-categories, three-categories, and four-categories) were compared to determine which best reflected the influence of sex and training form (HIIT vs. HIPT). Regression analyses (simple logistic regression for two-categories type, and multinomial logistic regression for three-categories and four-categories types) were performed using classification outcome as the dependent variable and sex, form, and their interaction as predictors. Model performance and goodness-of-fit for logistic regressions were evaluated using statistical criteria such as Akaike Information Criterion (AIC), and pseudo R^2^ (Nagelkerke) values. *P*-values of the sex and form and interaction term was analysed to assess the significance of the predictors. For the best fitted regression model descriptive statistics of the anthropometric measurements for categories of the participants depended on responses were calculated: mean, standard deviation and –95%CI. The same descriptive statistics for the intensity of the effort in subsequent sessions were calculated and repeated measures analysis of variance (RM ANOVA) was performed to study the differences. Bonferroni tests were applied for detailed comparisons. To identify distinct intensity response patterns over the 8-week intervention, a latent class mixed model (LCMM) was employed. The model was applied to the 16-session intensity trajectories (expressed as %HRmax), treating session as the time variable and intensity as the repeated outcome. Linear, quadratic, and cubic time effects were tested to determine the best-fitting model for latent class extraction. The optimal number of latent classes (i.e., intensity trajectory profiles) was determined by evaluating model fit indices including the Bayesian Information Criterion (BIC), Akaike Information Criterion (AIC), and entropy. Trajectory classes were not defined using predefined intensity thresholds or cut-off values; rather, they emerged from the LCMM as data-driven groupings of participants with similar longitudinal intensity patterns. Once latent classes were identified, participants were assigned to their most likely class based on posterior probabilities. Sex was included as a covariate in the LCMM to account for biological differences in physiological adaptation, in line with auxological and anthropometric considerations. Training form (HIIT vs. HIPT) was not included as a grouping variable during latent class extraction because the objective was to identify naturally occurring intensity trajectories based on the achieved exercise intensity itself, irrespective of intervention allocation. In addition, the available sample size limited the feasibility of fitting more complex stratified trajectory models. The potential influence of training form was subsequently examined in the regression analyses. Subsequently, the distribution of responders and non-responders (as defined by the binary Bayesian classification) was examined across the identified intensity trajectory classes using chi-square tests. Additional logistic regression models were used to explore whether trajectory class membership, sex, and their interaction predicted responder status. To investigate the relationship between individuals classified as responders (Rs) or non-responders (NRs) under the binary Bayesian approach and their assignment to latent classes derived from intensity trajectories, we used the best-fitting LCMM model as determined by the combination of AIC, BIC, and entropy values. The participants' binary classification (Rs vs. NRs) was cross-tabulated with their class membership from the selected LCMM model (e.g., 3-class model). A contingency table was created, and each cell included both absolute frequencies and row percentages. A chi-square test of independence was performed to examine the association between the two classification systems (being responder and. Additionally, a mosaic plot was used to visualize the joint distribution of Rs and NRs across the LCMM-derived classes, with color coding (green for Rs and red for NRs) and percentage annotations to support interpretation. To further explore the factors associated with being classified as a responder (Rs) or non-responder (NRs) under the Bayesian binary model, a generalized linear model (GLM) with binomial distribution was fitted. The dependent variable was responder status, while the predictors included intensity trajectory class (pattern, as derived from the LCMM analysis), sex, and training form (HIIT vs. HIPT). The model tested whether any of these factors significantly predicted the likelihood of being a responder. To investigate the extent to which changes in systolic (*Δ*SBP) and diastolic (*Δ*DBP) blood pressure were explained by intensity adaptation patterns (trajectory class), sex, and intervention form (HIIT vs. HIPT), two separate multiple linear regression models were conducted. In each model, *Δ*SBP and *Δ*DBP served as dependent variables, while trajectory class (categorical, with class 1 as reference), sex (male/female), and form (HIIT/HIPT) were included as predictors. Model fit was assessed using R^2^ and *F*-tests for overall significance. Standardized regression coefficients (*β*), standard errors, t-values, and corresponding *p*-values were reported for each predictor. In addition, partial eta squared (*η*^2^ₚ) was calculated to estimate the proportion of variance explained by each individual predictor, providing insight into the relative contribution of each factor. The significance level for all statistical tests and procedures was set at an *α*-value of 0.05. Calculations were conducted using RStudio 2025.05.0 + 496 (2009-2024 Posit Software, PBC).

## 3 Results

**Figure 1:**
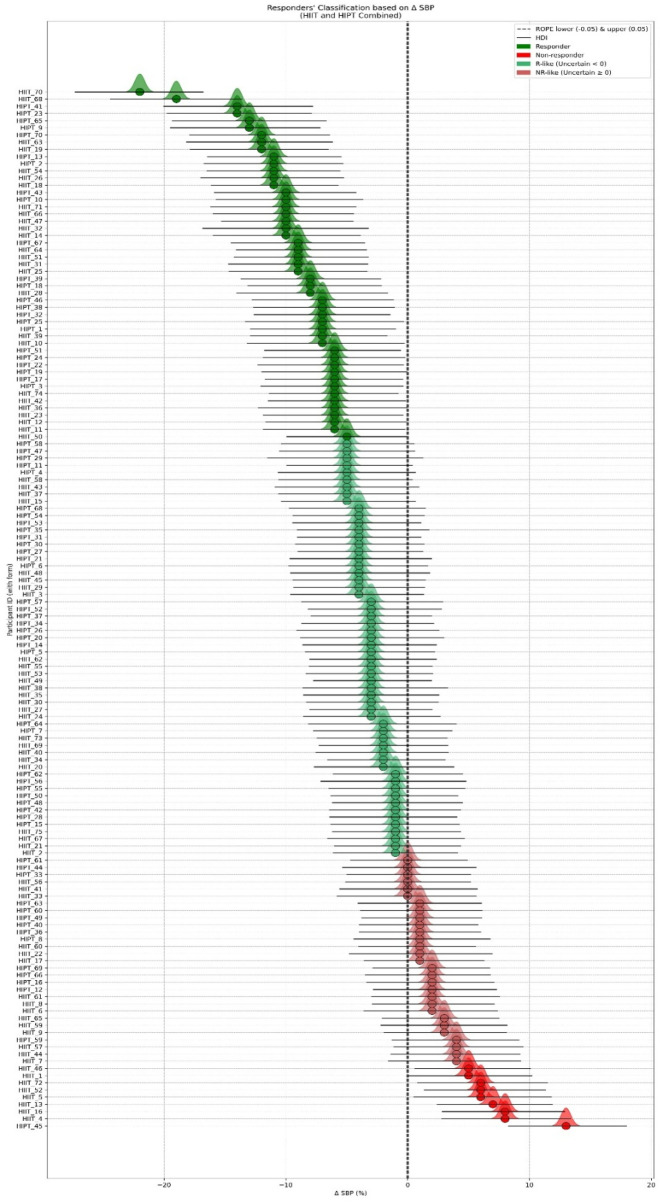
Overview of responder classification using the highest density interval (HDI) and region of practical equivalence (ROPE) method. The graph displays the individual changes in systolic blood pressure (*Δ*SBP) for each participant. Black dots represent the observed change for each individual, while the colored curves show the simulated normal distributions derived from the estimated measurement error around the observed *Δ*FAT value. Horizontal black lines represent the 89% HDI for each participant. Vertical dashed lines indicate the ROPE boundaries (−0.05 to 0.05), representing a range of practically negligible change.

**Figure 2:**
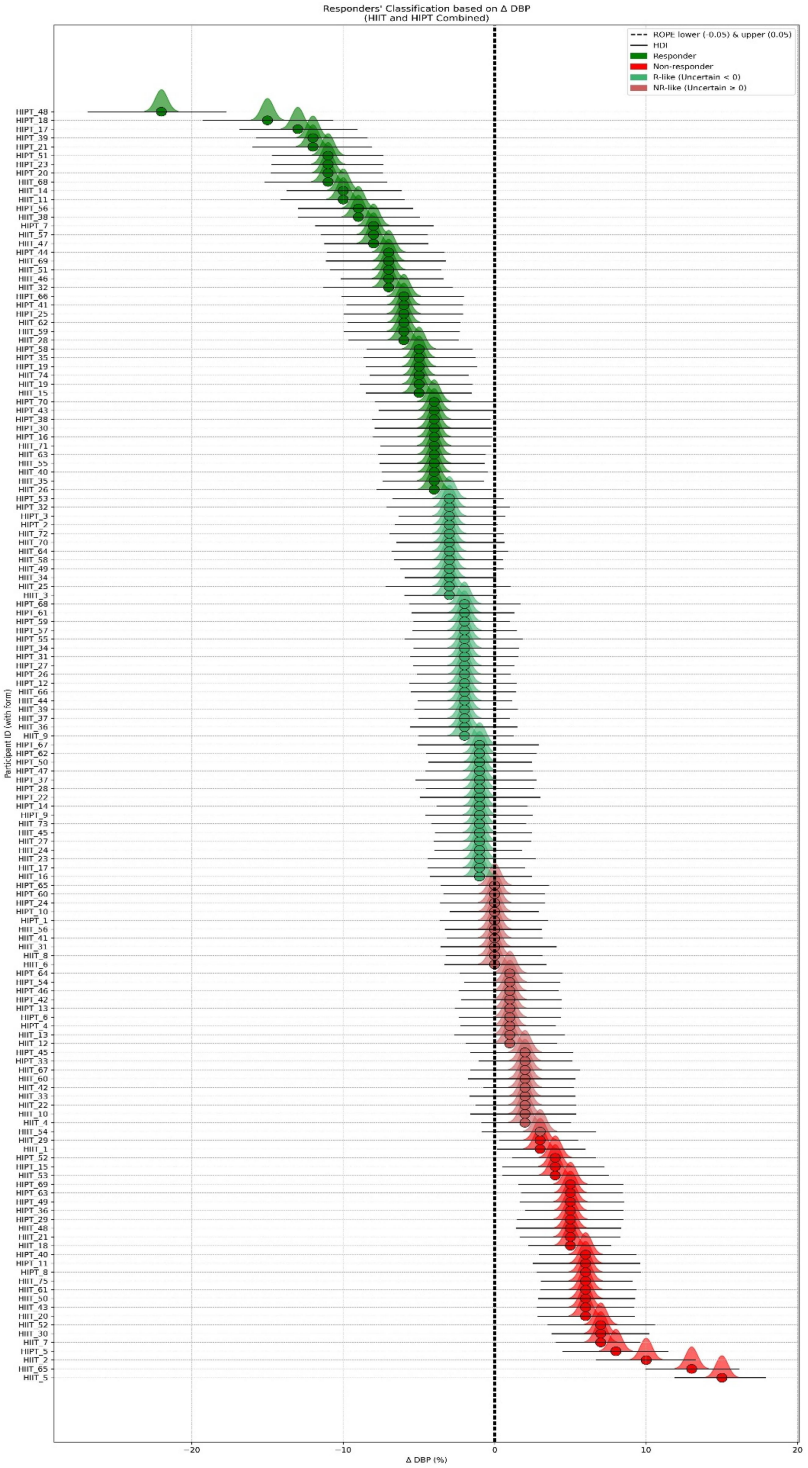
Overview of responder classification using the highest density interval (HDI) and region of practical equivalence (ROPE) method. The graph displays the individual changes in systolic blood pressure (*Δ*DBP) for each participant. Black dots represent the observed change for each individual, while the colored curves show the simulated normal distributions derived from the estimated measurement error around the observed *Δ*FAT value. Horizontal black lines represent the 89% HDI for each participant. Vertical dashed lines indicate the ROPE boundaries (−0.05 to 0.05), representing a range of practically negligible change.

Based on the Bayesian approach to classifying individual changes in systolic blood pressure (SBP) and diastolic blood pressure (DBP), participants were categorized into responders (Rs), non-responders (NRs), or uncertain cases (Figures 1, 2). For SBP, in the three-categories type, 30.4% (*n* = 44) were classified as responders, 63.4% (*n* = 92) as uncertain, and 6.2% (*n* = 9) as non-responders. For DBP 30.4% (*n* = 44) were classified as responders, 50.3% (*n* = 73) as uncertain, and 19.3% (*n* = 28) as non-responders.

A more nuanced four-categories classification distinguished uncertain cases into those showing improvement (Rs-like) or deterioration (NRs-like). For SBP, this resulted in 31.7% (*n* = 46) responders, 42.1% (*n* = 61) R-like, 20.0% (29) NR-like, and 6.2% (*n* = 9) non-responders. For DBP, there were 30.3% (*n* = 44) responders, 30.3% (*n* = 44) R-like, 20.0% (*n* = 29) NR-like, and 19.3% (*n* = 28) non-responders.

For subsequent binary analyses, a simplified classification was adopted by merging R-like participants with responders, and NR-like participants with non-responders. This binary model for SBP yielded 73.8% (*n* = 107) in the Rs group (body height: 170.35 ± 8.20 cm, body weight: 60.63 ± 10.52 kg) and 26.2% (*n* = 38) in the NRs group (body height: 168.65 ± 8.42 cm, body weight: 61.11 ± 12.37 kg). For DBP the frequencies and numbers were 60.7% (*n* = 88) in the Rs (body height: 169.42 ± 8.31 cm, body weight: 60.61 ± 11.09 kg) group and 39.3% (*n* = 57) in the NRs group (body height: 170.66 ± 8.21 cm, body weight: 60.99 ± 10.94 kg).

Those three classification types were compared for both SBP and DBP outcomes. As shown in Table 1, the binary type demonstrated the lowest AIC for both SBP (167.3) and DBP (197.9), indicating the best fit with the least complexity. Although the 4-categories type yielded slightly higher Nagelkerke R2 values for both outcomes (0.092 for SBP, 0.044 for DBP), this marginal gain did not outweigh the substantial increase in AIC and type complexity. For DBP, no predictors reached statistical significance in any type. For SBP, no main effects reached statistical significance across the compared classification approaches. Therefore, the 2-class type classification was selected for further analyses.

**Table 1:** Comparison of classification types for SBP and DBP in relation to sex and training form.

Outcome	Type	Categories	AIC	R^2^	Significant Predictors—main effects
SBP	Binary (GLM)	2	167.26	0.074	–
	Multinomial (MLR)	3	245.28	0.078	–
	Multinomial (MLR)	4	365.93	0.092	–
DBP	Binary (GLM)	2	197.92	0.041	–
	Multinomial (MLR)	3	308.80	0.035	–
	Multinomial (MLR)	4	413.21	0.044	–

*P* values were obtained from logistic regression models: binary generalized linear models (GLM, binomial distribution) for the 2-category classification and multinomial logistic regression (MLR) for the 3- and 4-category classifications.

Table 2 presents descriptive statistics of the intensity during all sixteen sessions (twice a week for 8 weeks) for all participants. The intensity during sixteen sessions have significantly changed what confirmed RM ANOVA results (*F* = 8.03, *p* < 0.001). However, each next session has not been significantly different than previous one (*p* > 0.500). The trend to decreasing intensity resulted in significantly lower intensity from 6th session in comparison to 1st to 5th sessions (*p* < 0.01). Lowest intensity was observed in 12th session, while slight increase was observed in four last sessions. It resulted in significant differences between 14th—16th sessions only in comparison to the 1st and 2nd session.

**Table 2:** Mean and standard deviation of relative intensity (%HR_max_) across 16 training sessions.

Session	Mean	−95%CI	+95%CI	SD
1	0.84	0.82	0.85	0.08
2	0.83	0.81	0.84	0.09
3	0.82	0.81	0.83	0.08
4	0.80	0.79	0.81	0.08
5	0.80	0.79	0.82	0.10
6	0.79	0.78	0.81	0.09
7	0.80	0.78	0.81	0.09
8	0.80	0.78	0.82	0.10
9	0.79	0.77	0.81	0.10
10	0.77	0.75	0.79	0.12
11	0.78	0.76	0.80	0.10
12	0.77	0.74	0.79	0.13
13	0.78	0.76	0.79	0.10
14	0.79	0.78	0.81	0.08
15	0.78	0.76	0.80	0.12
16	0.79	0.77	0.80	0.09

The overall *P* value was obtained using repeated-measures analysis of variance (RM ANOVA), and pairwise comparisons were performed using Bonferroni-adjusted *post hoc* tests.

**Figure 3:**
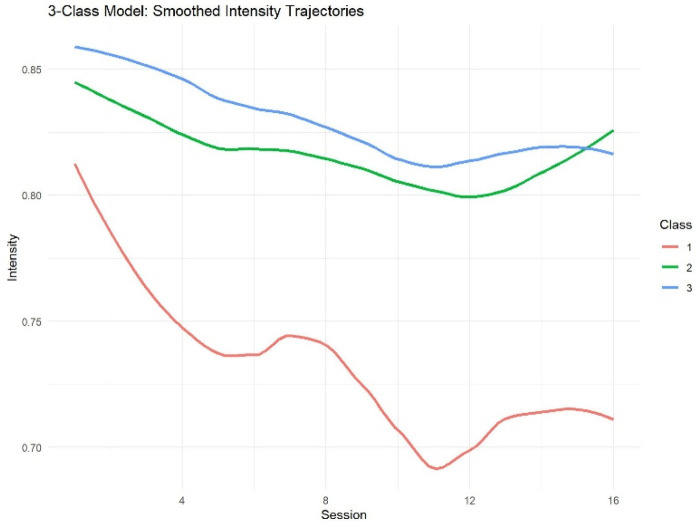
Smoothed intensity trajectories across 16 intervention sessions, identified via latent class mixed model (LCMM) with three latent profiles. Each trajectory represents the mean trend within a class based on session-by-session intensity values.

Latent class mixed models (LCMM) were estimated with two to five latent trajectories of exercise intensity over 16 sessions. Model fit was compared using Akaike Information Criterion (AIC), Bayesian Information Criterion (BIC), and entropy (Table 3). The 4-class model yielded the lowest AIC (–4,536.0), indicating the best overall fit. However, the 2-class model had the lowest BIC (–4,501.7), suggesting a more parsimonious solution. The 5-class model had the highest entropy (0.855), reflecting superior separation of latent trajectories. The 3-class model offered a compromise, with relatively low AIC/BIC and high entropy. Given the goal of balancing model fit and interpretability, the 3-classmodel was selected for subsequent analyses (Figure 3).

**Table 3:** Comparison of latent class mixed models (LCMM) with 2 to 5 classes based on AIC, BIC, and entropy.

Model	Number of Classes	AIC	BIC	Entropy
2-class	2	−4,528.47	−4,501.68	0.744
3-class	3	−4,524.41	−4,482.74	0.844
4-class	4	−4,535.97	−4,482.39	0.727
2-class	2	−4,528.47	24,501.68	0.744

No hypothesis-testing *P* values are reported in this table; model comparison was based on Akaike Information Criterion (AIC), Bayesian Information Criterion (BIC), and entropy.

Using the LCMM approach, three distinct trajectories of exercise intensity were identified across the 16-session intervention.

Class 3 (blue line) represents individuals who maintained consistently high intensity levels, showing a slight and shallow U-shaped pattern. After an initial decline, this group displayed regression in mid-sessions, followed by partial recovery and stabilization in later sessions, ending again at a relatively high intensity.

Class 2 (green line) followed a moderate-intensity declining trajectory. After a gradual decrease in intensity during the first two-thirds of the intervention, a modest recovery was observed in the final sessions. This pattern may reflect progressive fatigue or conservative pacing with only limited rebound.

Class 1 (red line) included participants with the lowest initial intensity and the steepest decline across sessions. Although some variability was observed toward the end, this group consistently showed the lowest intensity throughout, possibly reflecting poor adaptation or motivational decline. It shows L-shape like pattern, however with two local disturbances: peak between 7th–9th session and bottom in 11th session.

This classification highlights qualitatively distinct adjustment patterns during the intervention, which could be further explored in relation to physiological or psychological outcomes.

**Figure 4:**
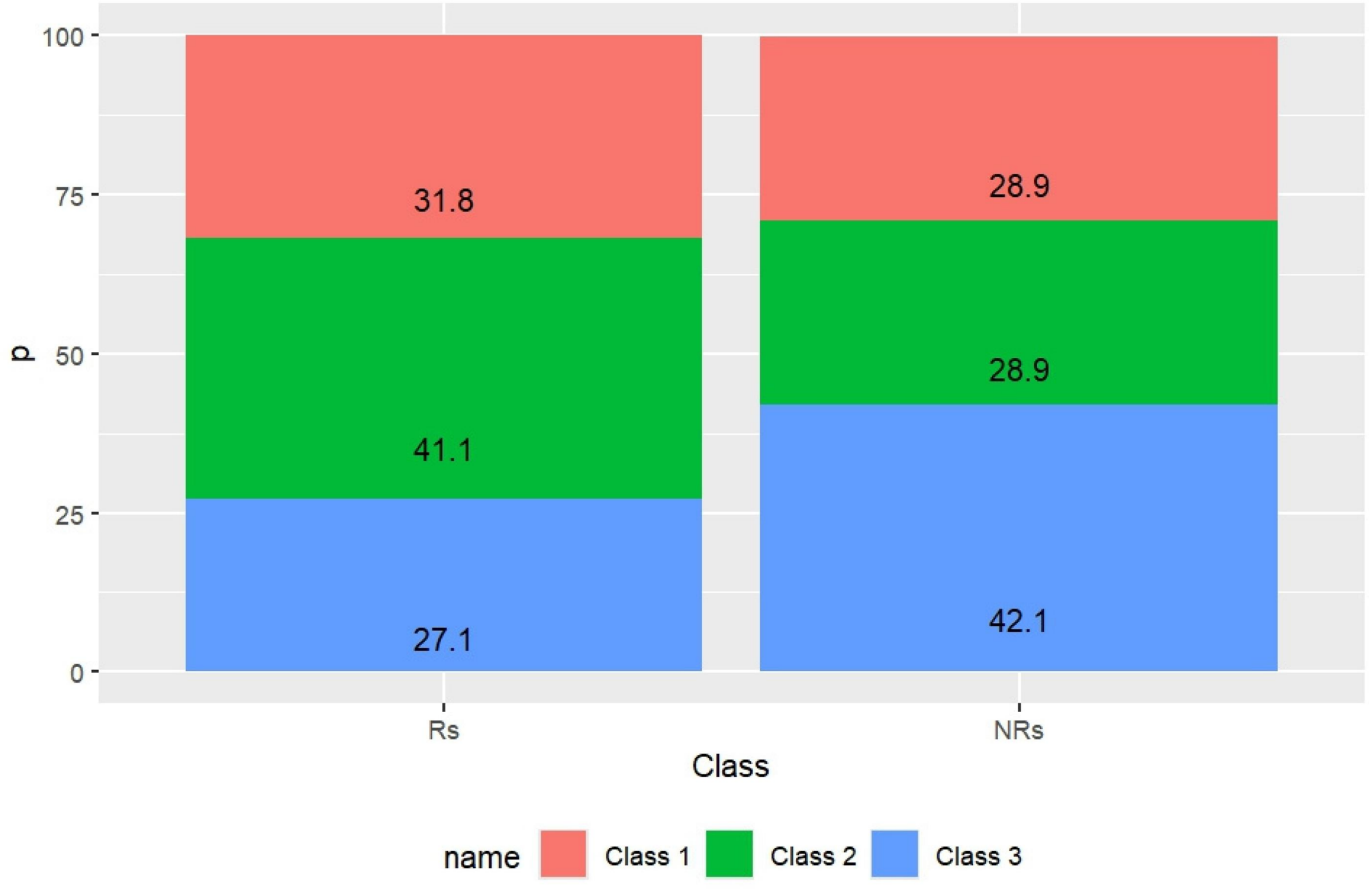
Mosaic plot illustrating the distribution of responders (Rs) and non-responders (NRs) across the three latent trajectory classes (class 1–3). Columns represent response classification (Rs vs. NRs), rows represent LCMM-derived intensity classes, and cell labels indicate within-row percentages.

The distribution of participants classified as Responders (Rs) and Non-Responders (NRs) across the three LCMM-derived intensity trajectory classes is presented in Figure 4. The most Rs were assigned to Class 2 (*n* = 44, 41.1%), while much less to Class 1 (*n* = 34, 31.8%) and Class 3 (*n* = 29, 27.1%). Notably, 42.1% of NRs were assigned to Class 3, while equal numbers (*n* = 11, 28.9%) to Class 1 and Class 2. Pearson's chi-squared test result indicated no statistically significant association between effects (response) and classification models (*χ*^2^ = 3.20, df = 2, *p* = 0.2017). As a robustness check—particularly due to relatively small cell sizes in some groups—Fisher's exact test was also computed. This test confirmed the lack of a statistically significant relationship (*p* = 0.1987).

The multiple logistic regression models (for both SBP and DBP) had a substantially lower AIC (162.69, 195.85, respectively) compared to simple models (all AIC >165, 195, respectively), supporting better overall fit. Simple logistic regression models for each predictor (trajectory class, sex, and training form) were not statistically significant on their own. However, the combined multiple logistic regression model revealed a significant effect, suggesting that these predictors may interact or jointly contribute to the likelihood of being classified as a responder (Table 4).

**Table 4:** Predictors of SBP and DBP responder status (logistic regression results).

Model	Variable	Predictor	Estimated	Std. Error	z-value	Pr(>|z|)	OR	(CI)	+95%CI	AIC
logistic	SBP	Trajectory (2)	1.35	0.64	2.09	0.036	3.86	1.08	14.03	
		Trajectory (3)	−1.66	0.65	−2.56	0.010	0.19	0.05	0.63	162.69
		Sex (M)	−2.29	0.79	−2.89	0.004	0.10	0.02	0.45	
		Form (HIPT)	−0.85	0.42	−2.03	0.043	0.43	0.18	0.97	
	DBP	Trajectory (2)	0.72	0.62	1.17	0.24	2.05	0.62	7.15	
		Trajectory (3)	−1.13	0.54	−2.09	0.04	0.32	0.11	0.91	195.88
		Sex (M)	−1.66	0.71	−2.34	0.02	0.19	0.04	0.74	
		Form (HIPT)	−0.71	0.38	−1.88	0.06	0.49	0.23	1.02	

*P* values were obtained from multiple logistic regression models (generalized linear models with binomial distribution).

In the multiple logistic regression model including all predictors simultaneously, trajectory class 3 (*β* = –1.66, *p* = 0.01, OR = 0.19) and sex (male) (*β* = –2.29, *p* = 0.02, OR = 0.10) were significantly associated with lower odds of being classified as a responder. Conversely, trajectory class 2 showed a positive trend (*β* = 1.35, *p* = 0.04, OR = 3.86), suggesting higher odds of response compared to class 1. The intervention form (HIPT) was also negatively associated with response (*β* = –0.85, *p* = 0.04, OR = 0.43).

In DBP-related multiply model, trajectory class 3 (*β* = –1.13, *p* = 0.04, OR = 0.32) and sex (male) (*β* = –1.66, *p* = 0.02, OR = 0.19) again showed significant negative associations with responder status. Intervention form (HIPT) was borderline non-significant (*p* = 0.06, OR = 0.49).

Trajectory class 2 (vs. class 1) significantly increased the odds of being a responder for SBP (OR = 3.86), suggesting that individuals following this pattern were nearly four times more likely to show a favorable blood pressure response. However, for DBP, the effect was minimal and not statistically meaningful (OR = 1.19). Trajectory class 3 was associated with a substantial decrease in the likelihood of being a responder in both outcomes. For SBP, the odds were 81% lower (OR = 0.19), and for DBP, 68% lower (OR = 0.32) compared to class 1. This suggests that the pattern characterizing class 3 may be less conducive to cardiovascular improvement. Sex played a strong and consistent role in both models. Male participants had significantly lower odds of being responders than females: 90% lower for SBP (OR = 0.10) and 81% lower for DBP (OR = 0.19). These findings point to potential sex-specific physiological adaptations or differential responses to the training stimulus. Regarding training form, participants in the HIPT group had lower odds of being classified as responders compared to those in the HIIT group—by 57% for SBP (OR = 0.43) and 51% for DBP (OR = 0.49), suggesting that HIIT may be more effective than HIPT in eliciting positive blood pressure responses in this adolescent population.

Multiple linear regression analyses were conducted to examine the effects of LCMM-derived trajectory class, sex, and intervention form (HIPT vs. HIIT) on changes in systolic (*Δ*SBP) and diastolic (*Δ*DBP) blood pressure (Table 5).

**Table 5:** Predictors of amount of change (*Δ*) in SBP and DBP (multiple linear regression results).

Model	Variable	Predictor	Estimated	Std. Error	t-value	Pr(>|t|)	F	*p*-value	*η*^2^_p_	R^2^
linear	SBP	Trajectory (2)	−4.84	1.53	−3.17	0.002				
		Trajectory (3)	−3.86	1.24	−3.11	0.002	5.027	<0.001	0.100	0.126
		Sex (M)	0.92	1.68	0.55	0.586			0.007	
		Form (HIPT)	−1.71	0.92	−1.86	0.064			0.020	
	DBP	Trajectory (2)	−5.35	1.50	−3.56	0.001				
		Trajectory (3)	−4.11	1.22	−3.36	0.001	6.757	<0.001	0.100	0.161
		Sex (M)	1.87	1.66	1.13	0.260			0.020	
		Form (HIPT)	−2.51	0.90	−2.78	0.006			0.050	

Predictor *P* values were obtained from multiple linear regression *t* tests, whereas model-level *P* values were obtained from the overall *F* test of the regression model.

For *Δ*SBP, the overall model was statistically significant (*F* = 5.03, *p* < .001), explaining 12.6% of the variance (R^2^ = 0.126). Compared to class 1, participants in class 2 showed significantly greater reductions in SBP (*B* = –4.84, *p* = .002), as did those in class 3 (*B* = –3.86, *p* = .002). The intervention form (HIPT) approached significance (*B* = –1.71, *p* = .064), suggesting a trend toward greater SBP reductions compared to HIIT. Sex was not a significant predictor (*B* = 0.92, *p* = .586). Partial eta-squared values indicated a moderate effect of trajectory (*η*^2^ₚ = 0.100) and small effects for other predictors.

For *Δ*DBP, the model was also significant (*F* = 6.76, *p* < .001), with a slightly higher explained variance (R^2^ = 0.161). Trajectory class remained a significant predictor: class 2 (*B* = –5.35, *p* = .001) and class 3 (*B* = –4.11, *p* = .001) both showed greater DBP reductions relative to class 1. Additionally, HIPT was associated with significantly greater DBP reductions (*B* = –2.51, *p* = .006), while sex remained non-significant (*B* = 1.87, *p* = .260). Partial eta-squared values again highlighted the strongest effect for trajectory (*η*^2^ₚ = 0.100), followed by intervention form (*η*^2^ₚ = 0.050).

## 4 Discussion

The primary aim of this study was to determine whether distinct intensity trajectory patterns observed during an eight-week school-based HIIT and HIPT intervention were associated with blood pressure (BP) adaptation in adolescents. The results confirmed the presence of three qualitatively different effort profiles: a low and progressively declining pattern (Class 1), a moderately high but steadily declining pattern (Class 2), and a U-shaped trajectory with an initial drop and partial recovery (Class 3). These findings support previous evidence suggesting that not only the overall training dose but also the temporal structure of exercise intensity may critically influence health outcomes (11, 23, 30). However, it is difficult to directly compare our findings with previous studies, as very few investigations have examined the trajectory of exercise load across an intervention in sufficient depth. Most prior research has focused on average or cumulative measures of training intensity, rather than on the dynamic patterns of effort over time (8, 10, 11, 16). Thus, the present study provides novel insights by highlighting how distinct intensity trajectories may differentially influence blood pressure adaptation in adolescents.

From a practical perspective, the present findings suggest that monitoring the average intensity of an intervention may provide an incomplete description of the exercise stimulus received by individual students. Session-by-session heart rate monitoring may help identify participants whose relative exercise intensity remains consistently low or declines substantially throughout the program. In school settings, such information could encourage teachers to review exercise selection, work-to-rest structure, instructions, or participant engagement. Nevertheless, the present study did not test a trajectory-based adjustment strategy, and the identified patterns should not yet be treated as thresholds for modifying individual exercise programs. The findings for Class 3 require a more nuanced interpretation. Participants in this class showed larger adjusted mean reductions in both SBP and DBP than those in Class 1, but they also had lower odds of meeting the binary response criterion. These results are not directly contradictory because the two analyses addressed different aspects of adaptation. Whereas the regression models evaluated average blood pressure changes at the group level, responder classification was based on an individual threshold approach that incorporated measurement uncertainty. Consequently, a trajectory class may exhibit larger mean reductions while simultaneously containing fewer individuals who satisfy the responder criterion. This apparent discrepancy highlights the distinction between population-level mean changes and individual-level responder classification, which should be viewed as complementary rather than interchangeable indicators of adaptation.

Importantly, logistic regression analyses indicated that participants assigned to Class 2 were almost four times more likely to be classified as responders for SBP compared to those in Class 1, whereas no corresponding association was observed for DBP. This finding suggests that the moderate-intensity, gradually declining trajectory identified in Class 2 may still be compatible with a favorable SBP response. However, the confidence interval around this estimate was relatively wide, and the result should therefore be interpreted cautiously. Moreover, the association was observed in an adjusted model and does not demonstrate that trajectory membership itself caused the blood pressure response. Nevertheless, the finding remains consistent with the broader concept that the magnitude and temporal distribution of the training stimulus may influence cardiovascular adaptation (11, 31, 32).

Interestingly, HIPT was linked to greater reductions in DBP, although it was simultaneously associated with lower odds of being classified as a responder. This divergence may reflect greater inter-individual variability in response to the intervention rather than a consistently less favorable effect of HIPT. Such heterogeneity is consistent with the concept of inter-individual variability in response to exercise training, according to which apparently similar exercise stimuli may produce markedly different physiological adaptations across participants (8, 33). Because vascular function, autonomic regulation, and acute hemodynamic responses were not assessed, the mechanisms underlying this finding cannot be determined from the present data. Another notable finding was the significant role of sex. Male participants were less likely to be classified as responders for both SBP and DBP, particularly in logistic regression models. In contrast, sex was not significantly associated with the magnitude of continuous SBP or DBP change. Thus, the role of sex depended on how blood pressure adaptation was operationalized. Previous studies have suggested that adolescent girls may exhibit greater sensitivity to vascular adaptations following high-intensity exercise, potentially due to sex-specific hormonal responses and endothelial reactivity (34, 35). These results emphasize the need for sex-specific considerations in the design of school-based training interventions. However, these mechanisms were not examined in the present study, and the observed associations should therefore be interpreted cautiously. Age was not included as a predictor because the participants represented a narrow age cohort, and the present study was not designed to test age-related differences in adaptation.

It is also noteworthy that while chi-square tests did not reveal significant associations between responder status and trajectory class, regression analyses demonstrated significant class-related associations after sex and training form were included as covariates. This difference may be explained by the fact that the chi-square test evaluated the unadjusted distribution of response categories across classes, whereas the regression model estimated the association of trajectory class with response after accounting for sex and intervention format. Regression modelling can therefore reveal adjusted associations that may not be evident in simple univariate comparisons (35, 36). The regression model did not test interactions between these predictors; therefore, the findings should be interpreted as adjusted associations rather than evidence of interactive effects.

The interpretation of these findings also depends on the definition of response. The primary Bayesian classification distinguished responders, non-responders, and uncertain cases. For the binary analyses, uncertain cases were subsequently assigned to responder-like or non-responder-like groups according to the direction of their observed change. Therefore, the binary outcome should not be interpreted as equivalent to a strict comparison between definite responders and definite non-responders. Alternative response definitions or thresholds could result in different class distributions and regression estimates.

Given the relatively limited sample size and the auxiliary nature of the responder-classification analysis, subgroup analyses were not conducted separately by sex or training form, which should be considered a study limitation. However, sex was retained as a covariate in all regression models due to its known biological relevance, particularly in the context of auxological and physiological variability in blood pressure responses. The study also did not include measures of heart rate variability or other indicators of autonomic cardiovascular regulation. Consequently, it was not possible to determine whether differences in autonomic adaptation contributed to the observed variability in systolic and diastolic blood pressure responses. A further limitation is that the present analyses were conducted on a subset of participants from the intervention arm of the parent trial. Consequently, the sample size was smaller than that originally planned for the full randomized controlled trial, which may have reduced statistical power to detect smaller associations, particularly in subgroup and responder analyses. Future studies with larger samples should further explore potential sex-specific mechanisms of response and combine session-by-session exercise-intensity monitoring with measures of heart rate variability, resting heart rate, and vascular function to better characterize the mechanisms underlying individual blood pressure adaptation. In addition, future studies should investigate whether the structure and membership of intensity trajectories differ between HIIT and HIPT interventions by incorporating training modality directly into trajectory modelling.

## 5 Conclusions

Using a Bayesian framework, we identified distinct proportions of responders (Rs), non-responders (NRs), and uncertain cases in both SBP and DBP outcomes. While these classifications were examined across latent intensity trajectories derived from LCMM, no significant association emerged between responder status and trajectory class, suggesting that favorable blood pressure adaptations may occur through diverse intensity patterns, potentially shaped by other intra- or inter-individual factors.

In contrast, logistic regression analyses revealed that intensity trajectory and sex jointly predicted responder status: individuals in Class 3 and males were less likely to be SBP responders, while male sex also significantly reduced the odds of DBP response. Furthermore, multiple linear regression models confirmed that intensity trajectories significantly predicted the magnitude of SBP and DBP changes, independent of sex and intervention type. Participants in Class 2 and 3 experienced greater reductions in both SBP and DBP, with trajectory class contributing moderate effect sizes. Although the HIPT form was associated with greater DBP reductions, sex showed no independent effect on continuous outcomes.

These findings show that adolescents may follow distinct exercise-intensity trajectories during school-based high-intensity interventions and that trajectory membership may be associated with blood pressure adaptation. Session-by-session monitoring may therefore complement conventional measures of average exercise intensity. However, the present findings are exploratory and do not establish a ready-to-use method for individualizing school-based exercise programs. Further research should determine whether modifying an intervention in response to an unfavorable intensity trajectory improves subsequent health outcomes.

## Data Availability

The raw data supporting the conclusions of this article will be made available by the authors, without undue reservation.

## References

[1] Hardy ST Urbina EM. Blood pressure in childhood and adolescence. Am J Hypertens. (2021) 34(3):242–9. 10.1093/ajh/hpab004 33821942 PMC8022980

[2] Lim SS Vos T Flaxman AD Danaei G Shibuya K Adair-Rohani H. A comparative risk assessment of burden of disease and injury attributable to 67 risk factors and risk factor clusters in 21 regions, 1990–2010: a systematic analysis for the global burden of disease study 2010. Lancet. (2012) 380:2224–60. 10.1016/S0140-6736(12)61766-8 23245609 PMC4156511

[3] Danaei G Ding EL Mozaffarian D Taylor B Rehm J Murray CJ. The preventable causes of death in the United States: comparative risk assessment of dietary, lifestyle, and metabolic risk factors. PLoS Med. (2009) 6:e1000058. 10.1371/journal.pmed.1000058 19399161 PMC2667673

[4] Popowczak M Rokita A Koźlenia D Domaradzki J. The high-intensity interval training introduced in physical education lessons decreases systole in high blood pressure adolescents. Sci Rep. (2022) 12:1974. 10.1038/s41598-022-06017-w Erratum in Sci. Rep. 2022, 12, 3397. 35132123 PMC8821617

[5] Eddolls WTB McNarry MA Stratton G Winn CON Mackintosh KA. High-intensity interval training interventions in children and adolescents: a systematic review. Sports Med. (2017) 47:2363–74. 10.1007/s40279-017-0753-8 28643209 PMC5633633

[6] Weston KL Azevedo LB Bock S Weston M George KP Batterham AM. Effect of novel, school-based high-intensity interval training (HIT) on cardiometabolic health in adolescents: project FFAB (fun fast activity blasts) – an exploratory controlled before-and-after trial. PLoS One. (2016) 11(8):e0159116. 10.1371/journal.pone.0159116 27486660 PMC4972319

[7] Zapata-Lamana R Cigarroa Cuevas I Fuentes V Soto Espindola C Parrado Romero E Sep-lveda C. HIITing health in school: can high intensity interval training be a useful and reliable tool in a school-based environment? A systematic review. Int J Sch Health. (2019) 6(3):1–10. 10.5812/intjsh.89829

[8] Bonafiglia JT Rotundo MP Whittall JP Scribbans TD Graham RB Gurd BJ. Inter-Individual variability in the adaptive responses to endurance and sprint interval training: a randomized crossover study. PLoS One. (2016) 11(12):e0167790. 10.1371/journal.pone.0167790 27936084 PMC5147982

[9] Andrade-Mayorga O Martínez-Maturana N Salazar LA Díaz E. Physiological effects and inter-individual variability to 12 weeks of high intensity-interval training and dietary energy restriction in overweight/obese adult women. Front Physiol. (2021) 12:713016. 10.3389/fphys.2021.713016 34393829 PMC8358598

[10] Domaradzki J Koźlenia D Popowczak M. The prevalence of responders and non-responders for body composition, resting blood pressure, musculoskeletal, and cardiorespiratory fitness after ten weeks of school-based high-intensity interval training in adolescents. J Clin Med. (2023) 12(13):4204. 10.3390/jcm12134204 37445239 PMC10342639

[11] Hecksteden A Kraushaar J Scharhag-Rosenberger F Theisen D Senn S Meyer T. Individual response to exercise training—a statistical perspective. J Appl Physiol. (2015) 118:1450–9. 10.1152/japplphysiol.00714.2014 25663672

[12] Atkinson G Williamson P Batterham AM. Issues in the determination of “responders” and “non-responders” in physiological research. Exp Physiol. (2019) 104:1215–25. 10.1113/EP087022 31116468

[13] Dankel SJ Bell ZW Spitz RW Wong V Viana RB Chatakondi RN. Assessing differential responders and mean changes in muscle size, strength, and the crossover effect to two distinct resistance training protocols. Appl Physiol Nutr Metab. (2020) 45:463–70. 10.1139/apnm-2019-0597 31553889

[14] Akubat I Patel E Barrett S Abt G. Methods of monitoring the training and match load and their relationship to changes in fitness in professional youth soccer players. J Sports Sci. (2012) 30(14):1473–80. 10.1080/02640414.2012.712711 22857397

[15] Stagno KM Thatcher R van Someren KA. A modified TRIMP to quantify the in-season training load of team sport players. J Sports Sci. (2007) 25(6):629–34. 10.1080/02640410600811817 17454529

[16] Frank HR Mulder H Sriram K Santanam TS Skinner AC Perrin EM. The dose-response relationship between physical activity and cardiometabolic health in young adults. J Adolesc Health. (2020) 67(2):201–8. 10.1016/j.jadohealth.2020.04.021 32571756 PMC11218041

[17] Chang X Wang Z Guo H Xu Y Ogihara A. Effect of physical activity/exercise on cardiorespiratory fitness in children and adolescents with type 1 diabetes: a scoping review. Int J Environ Res Public Health. (2023) 20(2):1407. 10.3390/ijerph20021407 36674162 PMC9860959

[18] Gibala MJ Jones AM. Physiological and performance adaptations to high-intensity interval training. Nestle Nutr Inst Workshop Ser. (2013) 76:51–60. 10.1159/000350256 23899754

[19] Ma Q Pei G Meng L. Inverted U-shaped curvilinear relationship between challenge and intrinsic motivation: evidence from event-related potentials. Front Neurosci. (2017) 11:131. 10.3389/fnins.2017.00131 28400712 PMC5368271

[20] Chang YK Chu CH Wang CC Wang YC Song TF Tsai CL. Dose-response relation between exercise duration and cognition. Med Sci Sports Exerc. (2015) 47:159–65. 10.1249/MSS.0000000000000383 24870572

[21] Wang D Zhou C Zhao M Wu X Chang YK. Dose-response relationships between exercise intensity, cravings, and inhibitory control in methamphetamine dependence: an ERP study. Drug Alcohol Depend. (2016) 161:331–9. 10.1016/j.drugalcdep.2016.02.023 26946990

[22] Sian TS Inns TB Gates A Doleman B Bass JJ Atherton PJ. Equipment-free, unsupervised high intensity interval training elicits significant improvements in the physiological resilience of older adults. BMC Geriatr. (2022) 22:529. 10.1186/s12877-022-03208-y Erratum in BMC Geriatr. 2022, 22, 926. 35761262 PMC9238013

[23] Bauer N Sperlich B Holmberg HC Engel FA. Effects of high-intensity interval training in school on the physical performance and health of children and adolescents: a systematic review with meta-analysis. Sports Med Open. (2022) 8:50. 10.1186/s40798-022-00437-8 35403996 PMC9001771

[24] Domaradzki J Popowczak M Kochan-Jacheć K Szkudlarek P Murawska-Ciałowicz E Koźlenia D. Effects of two forms of school-based high-intensity interval training on body fat, blood pressure, and cardiorespiratory fitness in adolescents: randomized control trial with eight-week follow-up – the PEER-HEART study. Front Physiol. (2025) 16:1530195. 10.3389/fphys.2025.1530195 40265155 PMC12011756

[25] Faul F Erdfelder E Lang AG Buchner A. G*power 3: a flexible statistical power analysis program for the social, behavioral, and biomedical sciences. Behav Res Methods. (2007) 39(2):175–91. 10.3758/BF03193146 17695343

[26] Nasir K Ziffer JA Cainzos-Achirica M Ali SS Feldman DI Arias L. The Miami heart study (MiHeart). Am J Prev Cardiol. (2021) 7:100202. 10.1016/j.ajpc.2021.100202 34611641 PMC8387278

[27] Duncombe SL Barker AR Price L Walker JL Koep JL Woodforde J. Was it a HIIT? A process evaluation of a school-based high-intensity interval training intervention. Int J Behav Nutr Phys Act. (2024) 21(1):49. 10.1186/s12966-024-01599-2 38684991 PMC11059682

[28] Karvonen MJ Kentala E Mustala O. The effects of training on heart rate: a longitudinal study. Ann Med Exp Biol Fenn. (1957) 35(3):307–15. 13470504

[29] Maturana FM Soares RN Murias JM Schellhorn P Erz G Burgstahler C. Responders and non-responders to aerobic exercise training: beyond the evaluation of VO_2_max. Physiol Rep. (2021) 9(14):e14951. 10.14814/phy2.14951 34409753 PMC8374384

[30] Vollaard NBJ Metcalfe RS. Research into the health benefits of sprint interval training should focus on protocols with fewer and shorter sprints. Sports Med. (2017) 47:2443–51. 10.1007/s40279-017-0727-x 28391489 PMC5684281

[31] Montero D Lundby C. Refuting the myth of non-response to exercise training: “non-responders” do respond to higher dose of training. J Physiol. (2017) 595:3377–87. 10.1113/JP273480 28133739 PMC5451738

[32] Cornelissen VA Smart NA. Exercise training for blood pressure: a systematic review and meta-analysis. J Am Heart Assoc. (2013) 2:e004473. 10.1161/JAHA.112.004473 23525435 PMC3603230

[33] Mann TN Lamberts RP Lambert MI. High responders and low responders: factors associated with individual variation in response to standardized training. Sports Med. (2014) 44:1113–24. 10.1007/s40279-014-0197-3 24807838

[34] DuPont JJ Kenney RM Patel AR Jaffe IZ. Sex differences in mechanisms of arterial stiffness. Br J Pharmacol. (2019) 176(21):4208–25. 10.1111/bph.14624 30767200 PMC6877796

[35] Domaradzki J Koźlenia D Popowczak M. Sex moderated mediation of the musculoskeletal fitness in relationship between high-intensive interval training performing during physical education classes and cardiorespiratory fitness in healthy boys and girls. BioMed Res Int. (2022) 2022:8760620. 10.1155/2022/8760620 35083335 PMC8786534

[36] Atkinson G Batterham AM. True and false interindividual differences in the physiological response to an intervention. Exp Physiol. (2015) 100:577–88. 10.1113/EP085070 25823596

